# Therapeutic Effect of Seawater Pearl Powder on UV-Induced Photoaging in Mouse Skin

**DOI:** 10.1155/2021/9516427

**Published:** 2021-12-09

**Authors:** Siyin Han, Delun Huang, Taijin Lan, Yongpei Wu, Yingbiao Wang, Jiying Wei, Weiyuan Zhang, Yuanyang Ou, Qiangqiang Yan, Peng Liu, Zhenxing Chen, Jiang Lin

**Affiliations:** School of Basic Medicine, Guangxi University of Chinese Medicine, Nanning, China

## Abstract

The objective of this study was to investigate the therapeutic effect of seawater pearl powder (SPP) on ultraviolet (UV) irradiation-induced photoaging in mouse skin. The protein and trace elements in SPP were detected by liquid chromatography-mass spectrometry, atomic fluorescence spectrometry, and inductively coupled plasma-atomic emission spectrometry. The effect of SPP on treating skin damage resulting from UV-induced photoaging was observed by gross physical appearance and histopathological analysis. Oxidative stress and melanin synthesis were analyzed using biochemical method. Western blotting was applied to analyze the phosphorylation and expression levels of matrix metalloproteinase-1 (MMP-1), collagen I, and proteins involved in the mitogen-activated protein kinase (MAPK) signaling pathways (p38, ERK, and JNK). The results show that SPP has a significant therapeutic effect on UV-induced photoaging of skin and improves and restores appearance and tissue structure of mouse skin. The major mechanism may be related to reduction of expression level of MMP-1 and enhancement of collagen I production via inhibition of MAPK signaling pathway after scavenging of excess reactive oxygen species (ROS) in the UV-induced photoaged skin of mice. Meanwhile, it may also be involved in reducing melanin content by inhibiting tyrosinase activity after scavenging excess ROS in the UV-induced photoaged skin of mice. Therefore, SPP could be a good substance to treat photoaging skin. Taking cost-effectiveness and efficacy into consideration, the optimal concentration of SPP for treating photoaging skin could be 100 mg/g.

## 1. Introduction

Skin photoaging is mainly caused by environmental factors, such as ultraviolet (UV) irradiation, smoking, and chemicals. Among these, UV irradiation, such as UVA and UVB, is the primary cause of accelerated photoaging, which disrupts the balance between collagen production and degradation resulting in functional collagen loss. Clinically, photoaging of skin begins with epidermal hyperplasia, followed by atrophic wrinkles, flabby nodules, irregular pigmentation, telangiectasia, and bulky skin pores [1]. Skin photoaging not only affects physical appearance and increases the psychological burden on patients, hindering normal work and social contact, but also has an etiological connection with the occurrence of benign skin tumors, “precancerous” lesions, and skin cancer [2].

Previous studies have shown that UV irradiation-induced reactive oxygen species (ROS) mediate the phosphorylation of protein kinases through the mitogen-activated protein kinase (MAPK) signaling pathway, which involves the upregulation of extracellular signal-regulated kinases (ERK), c-Jun amino terminal kinase (JNK), and p38 [3]. The activation of the MAPK signaling pathway directly results in phosphorylation of the activator protein-1 (AP-1) complex, which upregulates the expression of matrix metalloproteinases (MMPs) [4]. UV irradiation upregulates the expression of matrix metalloproteinase-1 (MMP-1) that initiates the degradation of type I and type III collagen [5], and the degraded collagen fragments produced by MMPs downregulate new collagen synthesis *in vitro* and *in vivo* [6]. In addition, melanin overproduction can be stimulated by UV irradiation [7]. The irregular pigmentation of photoaged skin is determined by the melanin content in the epidermis, and tyrosinase (TYR) is the main catalyst for melanin synthesis [8].

Pearl, as a precious Chinese herbal medicine, has been used for thousands of years. It is a natural inorganic-organic composite material with excellent properties, which is formed by biological self-assembly of calcium carbonate, trace organic matter (mainly protein), and some trace elements [9]. “The Pharmacopoeia of the People's Republic of China” clearly states that pearl could detoxify and invigorate the muscles, calm the nerves, clear the eyes, and eliminate turbidity. Shizhen Li believed that pearl beautifies the skin. The “Compendium of Materia Medica” mentions that when the pearl is painted on the surface of the skin, it makes the skin moist. It can also be used to remove skin spots, reduce phlegm, remove acne and toxins, and make the luster white. However, the antiphotoaging effect and underlying mechanism of action of pearl on skin have not yet been elucidated.

Therefore, in this study, the protein and trace elements in seawater pearl powder (SPP) were detected, and the skin photoaging animal model was established by using Institute of Cancer Research (ICR) mice. The appearance, tissue structure, oxidative stress, pigment precipitation index, and regulation of related proteins were analyzed to explore the effect and possible mechanism of action of SPP in treating skin photoaging.

## 2. Materials and Methods

### 2.1. Instruments and Chemicals

The instruments used were an Ultimate 3000 system capillary high performance liquid chromatography coupled with a *Q* Exactive™ Hybrid Quadrupole-Orbitrap™ Mass Spectrometer with an ESI nanospray source, inductively coupled plasma-atomic emission spectrometer (ICP-AES) (Thermo Fisher Scientific, Waltham, MA, USA); atomic fluorescence spectrometer (Beijing Jitian Instrument Co., Ltd., Beijing, China); self-made lamp box simulating solar light source: ultraviolet H-type lamp purchased from Philips Lighting Investment Co., Ltd. (Shanghai, China), which included one UVA lamp with power of 9 W and spectral wave of 364–366 nm and one UVB lamp with power of 9 W and spectral wave of 308–311 nm. Chemicals used were as follows: RIPA Lysis and Extraction Buffer was purchased from Thermo Fisher Scientific (Waltham, MA, USA). Trypsin was purchased from Promega (Madison, WI, USA). Determination kits for BCA protein concentration, superoxide dismutase (SOD), malondialdehyde (MDA), catalase (CAT), ROS, and TYR were purchased from Nanjing Jiancheng Bioengineering Institute (Nanjing, China); melanin standard products were purchased from Sigma (St. Louis, MO, USA); monoclonal antibodies, namely, anti-GAPDH, anti-MMP-1, anti-collagen I, anti-ERK, anti-JNK, anti-p38, anti-p-ERK, anti-p-JNK, and anti-p-p38, were purchased from Abcam (Milton, Cambridge, UK). Goat anti-rabbit IgG-horseradish peroxidase (HRP) secondary antibody was purchased from Aspen (Linden, Utah, USA).

### 2.2. Analysis of Proteins and Trace Elements in SPP

Protein analysis was as follows: firstly, RIPA Lysis and Extraction Buffer was added to the SPP sample to extract protein, then SDS-PAGE was performed, the corresponding strips were cut and decolorized, and the decolorized colloidal particles were hydrolyzed with trypsin. Then, the processed samples were analyzed by liquid chromatography-mass spectrometry to obtain the raw file of the original results of mass spectrometry, which was analyzed by software Maxquant (1.6.2.10) (Max Planck Institute of Biochemistry, Martins reed, Munich, Germany) to match the data. We obtained the identification results. Trace element analysis was as follows: we weighed 0.5 g SPP sample, put it into a microwave digestion tank, added 5 ml nitric acid, and put it into the microwave digestion instrument for microwave digestion. After digestion, we cooled it to room temperature and diluted it to 25 ml with ultrapure water to obtain the sample determination solution. The elements, Fe, Mn, Cu, Na, Mg, Zn, Se, As, and Hg, were determined by atomic fluorescence spectrometry. The elements, S, P, and Sr, were determined by ICP-AES.

### 2.3. Experimental Animals and Preparation of SPP Ointment

Female ICR mice (6–8 weeks old, 28–32 g) were purchased from Hunan Slake Jingda Experimental Animal Co., Ltd. (Changsha, China; permit number: SCXK (Xiang) 2019–0004). All animal procedures involved in this study complied with international ethical principles and the Guide for the Care and Use of Laboratory Animals.

SPP was purchased from Beihai Baozhulin Marine Technology Co., Ltd. (Beihai, China). SPP was added to vaseline and after stirring evenly, SPP ointment with concentrations of 50 mg/g, 100 mg/g, and 200 mg/g was prepared.

### 2.4. Grouping and Skin Photoaging Treatment in ICR Mice

ICR mice were randomly divided into the following groups: control group (CON), UV-induced group (UV), SPP low-dose group (SPPL), SPP medium-dose group (SPPM), and SPP high-dose group (SPPH), with 10 mice in each group. The hair over an area of 4.0 cm × 6.0 cm on the back of mice in each group was shaved with an electric razor. The UV group, SPPL group, SPPM group, and SPPH group were treated with 9W UVA and UVB lamps at regular times each day. The light source was simulated by placing UVA and UVB lamps above the exposed skin area of the back of mice for 2 hours at a distance of 20 cm; the irradiation range was 60 cm × 80 cm. Thus, the daily irradiation dose of UVA and UVB was about 13.5 J/cm^2^. The skin changes on the back of the mice were observed after daily UV irradiation. After 8 weeks of continuous irradiation, the skin of the UV group, SPPL group, SPPM group, and SPPH group developed wrinkles, thickening, roughness, and even damage. These results illustrated that the skin photoaging model was established. The CON group was not treated with any UV irradiation. Since the hair on the bare skin area of the back of mice would have continued to grow, the mice in each group needed to be shaved with an electric razor once per week.

### 2.5. Administration of Chemicals in ICR Mice

After the skin photoaging model was established in ICR mice, UV irradiation on the bare skin area of the back of mice was stopped. Then, the mice were given chemicals at regular times per day for 4 weeks. A layer of vaseline was smeared on the bare skin area of the back of mice in the CON group and the UV group; a layer of SPP ointment was applied to the bare skin area of the back of mice in the SPPL group (50 mg/g), SPPM group (100 mg/g), and SPPH group (200 mg/g).

### 2.6. Sampling and Index Detection

At the end of the experiment, the skin appearance changes of the mice were recorded by photography. Some skin tissue from the bare skin area of the back of mice in each group were fixed in 4% paraformaldehyde for making histopathological tissue sections. The other part of the skin tissue was stored at –80°C for determination of oxidative stress, pigment precipitation index, and related protein expression.

#### 2.6.1. Evaluation of Skin Histopathology

The fixed skin tissue sections were dehydrated in an ascending alcohol series (50% to 95% concentration). Dehydrated tissues were embedded in paraffin, sectioned into 5 *μ*m thick sections, and stained with hematoxylin and eosin (HE). Sections were then examined with a light microscope.

#### 2.6.2. Detection of Activities of SOD, CAT, TYR, and Contents of ROS and MDA by Biochemical Methods

The skin tissues of mice in each group, which were stored at –80°C, were homogenized with cooled Tris-HCl buffer at 1 : 5 (w/v), centrifuged at 4°C at 3,500 r/min for 10 min, and the supernatant was taken out for sample analysis. The activities of SOD, CAT, TYR, and the contents of ROS and MDA in skin tissues of each group were detected according to the operation instructions of the respective kits. The ROS content was expressed as the fluorescence intensity of soluble protein per mg. MDA content and SOD, CAT, and TYR activities were expressed in relative units of soluble protein per mg.

#### 2.6.3. Detection of Melanin Content by NaOH Dissolution Method

The skin tissues of mice in each group, which were stored at –80°C, were homogenized with cooled Tris-HCl buffer at 1 : 5 (w/v) and centrifuged at 4°C at 10,000 r/min for 10 min, the sediment was collected, 1 mol/L NaOH solution was added to completely dissolve the sediment, and then absorbance was measured at 475 nm. The standard substance of melanin was prepared into a solution, the standard curve was drawn, and the melanin content of skin was calculated [10].

#### 2.6.4. Detection of Protein Expression of MMP-1, Collagen I, and Phosphorylation Level of MAPK Signaling Pathway by Western Blot

The skin tissues of mice in each group, which were stored at –80°C, were homogenized with cooled protein extraction reagent at 1 : 10 (w/v). The homogenate was transferred to a centrifuge tube, vibrated and kept in an ice bath for 30 min, and then centrifuged at 4°C at 12,000 r/min for 5 min. The supernatant was collected, to which was added 5 × protein loading buffer of appropriate equivalent, and then immersed in boiling water at 100°C for 5 min as protein sample. The separation gel and concentrated gel were prepared, and then the protein samples were added to the sampling wells. Transfer membrane filter paper and methanol-activated PVDF membrane were prepared, and current was allowed to flow through the membrane at a constant rate of 300 mA. The transferred membrane was added into sealing solution and sealed for 1 hour at room temperature. The blocking solution was removed, and the monoclonal antibodies, namely, anti-GAPDH, anti-MMP-1, anti-collagen I, anti-ERK, anti-JNK, anti-p38, anti-p-ERK, anti-p-JNK, and anti-p-p38, were added after dilution with diluent for monoclonal antibodies and incubated at 4°C overnight. The diluted monoclonal antibodies were recovered and washed with TBST thrice, for 5 min each time. The diluted goat anti-rabbit IgG-HRP secondary antibody was added and incubated at room temperature for 30 min. TBST was used for washing four times on a shaking table at room temperature for 5 min each time. Fresh mixed ECL solution was added to the protein side of the membrane, which was then exposed in a dark room. The film was archived, analyzed, and scanned [11].

### 2.7. Statistical Analysis

Data of composition of various bacteria in the shrimp intestines are all presented as mean ± standard error (SE). Statistical analysis was performed using SPSS (version 17.0) (IBM, Armonk, NY, USA). One-way analysis of variance (ANOVA) and Least Significant Difference (LSD) tests were used to analyze the differences among the different treatment groups. A value of *p* < 0.05 was considered to be statistically significant. All images were generated with Origin 8.6 software (OriginLab, Northampton, MA, USA).

## 3. Results

### 3.1. Proteins and Trace Elements in SPP

**Figure 1:**
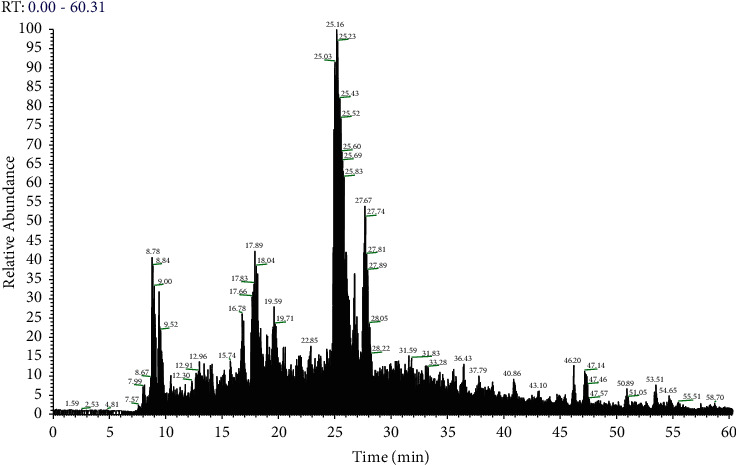
Total ion flow chromatogram of protein mass spectrometry of SPP.

After protein extraction and LC-MS/MS data collection of SPP, the raw file was generated. The total ion flow chromatogram of protein mass spectroscopy is shown in Figure 1. After searching the software Maxquant (1.6.2.10) database, a total of 178 proteins were identified. These proteins included many antioxidant enzyme-related proteins, such as sigma class glutathione S-transferase, glutathione peroxidase, glutathione S-transferase A, superoxide dismutase, glutathione synthase, catalase, GST class pi, GST 2, peroxiredoxin 6, GST 1, glutathione transferase, and superoxide dismutase [Cu Zn]. There were also some metal link-related proteins, such as metal binding protein, metal response element binding transcription factor-1, and metal response transcription factor 2, which may link metal elements in SPP to form metalloproteins. It is speculated that these proteins play a certain role in anti-skin photoaging. See annexure 1 for list of 178 proteins in SPP detected by protein mass spectrometry.  Table 1 shows only the top 20 proteins with high intensity in SPP.  Table 2 shows the contents of trace elements in SPP.

### 3.2. Effect of SPP on Skin Appearance of Photoaged Mice

**Figure 2:**
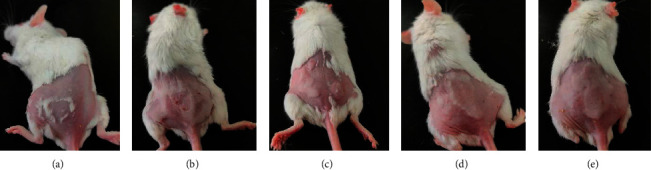
The skin photoaging model was established in mice after 8 weeks of continuous UV irradiation; (a) CON group, (b) UV group, (c) SPPL group, (d) SPPM group, and (e) SPPH group.

**Figure 3:**
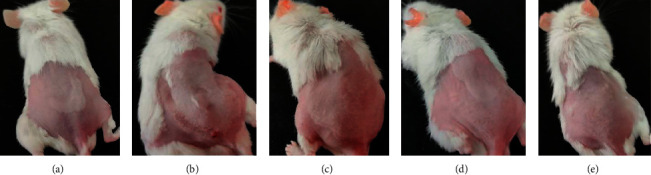
Effect of SPP on the appearance of photoaging skin in mice after the experiment; (a) CON group, (b) UV group, (c) SPPL group, (d) SPPM group, and (e) SPPH group.

After 8 weeks of continuous UV irradiation, the skin of CON group mice was smooth and thin, and subcutaneous blood vessels could be seen, showing flesh color. The skin of UV group, SPPL group, SPPM group, and SPPH group became damaged, rough, and thick; no subcutaneous blood vessels could be found, and skin color turned red. Thus, the skin photoaging models in the UV group, SPPL group, SPPM group, and SPPH group were established successfully in mice (Figure 2). Then, after 4 weeks of administering SPP ointment on the skin of photoaged mice, the experiment was terminated. The skin of CON group mice remained as healthy as before. The skin of UV group mice showed persistence of photoaging changes. The skin damage of SPPL group mice disappeared, but the skin was still rough, thick, and red. The skin damage of SPPM and SPPH groups disappeared, with significant amelioration of the rough, thick, and red skin appearance; the skin color of mice in the SPPH group was close to the CON group (Figure 3).

### 3.3. Effect of SPP on Tissue Structure of Photoaged Skin of Mice

**Figure 4:**
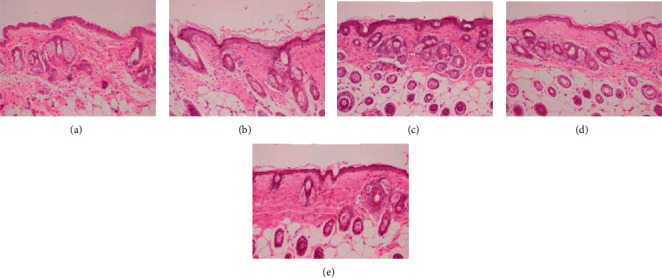
Effect of SPP on tissue structure of photoaging skin in mice after the experiment; (a) CON group, (b) UV group, (c) SPPL group, (d) SPPM group, and (e) SPPH group; H&E (×200), scale = 50 *μ*m.

The epidermis of mice in the CON group was compact and thin, with evident dermal papillae, connecting the dermis and epidermis closely, and the dermal reticulated layer was arranged tightly and evenly (Figure 4(a)). In the UV group mice, the structure of the epidermal layer was loose, the thickness increased, and there was fracture gully phenomenon. The dermal papillae had disappeared, and the reticulated layer was loose (Figure 4(b)). In the SPPL group mice, the epidermis was slightly thinner, but the structure was still loose (Figure 4(c)). In the SPPM group mice, the epidermis became significantly thinner, but the structure was still loose, and the dermal papillae were not evident (Figure 4(d)). In the SPPH group mice, the epidermis became significantly thinner, the dermal papillae recovered, and the dermal papillae were closely connected with the epidermis; this appearance was close to that of mice in the CON group (Figure 4(e)).

### 3.4. Effects of SPP on Contents of ROS and MDA and Activities of SOD and CAT in Photoaged Skin of Mice

**Figure 5:**
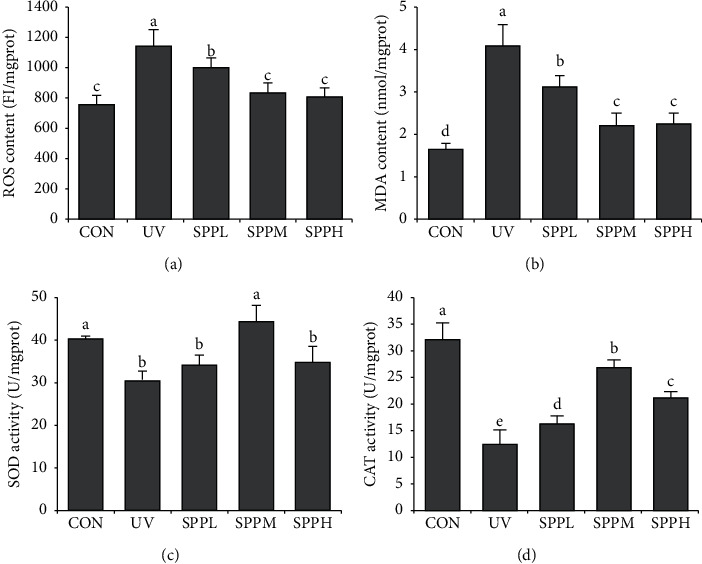
Effect of SPP on contents of ROS (a) and MDA (b) and activities of SOD (c) and CAT (d) in photoaging skin of mice after the experiment. Each bar represents the mean value from three repetitions with standard error (SE); different letters represent significant differences between the two groups.

Compared with the CON group, the contents of ROS and MDA in the skin of the UV group mice increased significantly (*p*< 0.05). Compared with the UV group, the contents of ROS and MDA in the skin of mice in the SPPL, SPPM, and SPPH groups decreased significantly (*p* < 0.05). Compared with the SPPL group, the contents of ROS and MDA in the skin of mice in the SPPM and SPPH groups decreased significantly (*p* < 0.05) (Figures 5(a) and 5(b)).

Compared with the CON group, the activities of SOD and CAT in the skin of mice in the UV group decreased significantly (*p*< 0.05). Compared with the UV group, the activity of SOD in the skin of mice in the SPPM group increased significantly (*p* < 0.05); the activity of CAT in the skin of mice in the SPPL, SPPM, and SPPH groups increased significantly (*p*< 0.05). Compared with the SPPL group, the activity of CAT in the skin of mice in the SPPM and SPPH groups increased significantly (*p*< 0.05). Compared with the SPPM group, the activity of CAT in the skin of mice in the SPPH group decreased significantly (*p* < 0.05) (Figures 5(c) and 5(d)).

### 3.5. Effect of SPP on TYR Activity and Melanin Content of Photoaged Skin of Mice

**Figure 6:**
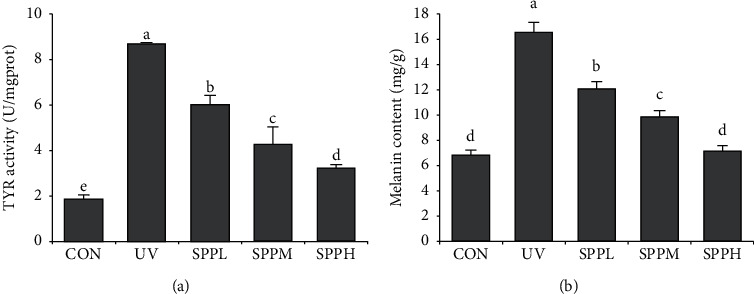
Effect of SPP on TYR activity (a) and melanin content (b) of photoaging skin in mice after the experiment; each bar represents the mean value from three repetitions with standard error (SE); different letters represent significant differences between the two groups.

Compared with the CON group, TYR activity and melanin content in the skin of mice in the UV group increased significantly (*p* < 0.05). Compared with the UV group, TYR activity and melanin content in the skin of mice in the SPPL, SPPM, and SPPH groups decreased significantly (*p* < 0.05). Compared with the SPPL group, TYR activity and melanin content in the skin of mice in the SPPM and SPPH groups decreased significantly (*p* < 0.05). Compared with the SPPM group, TYR activity and melanin content in the skin of the mice in the SPPH group decreased significantly (*p* < 0.05) (Figure 6).

### 3.6. Effect of SPP on Protein Expression of MMP-1 and Collagen I in Photoaged Skin of Mice

**Figure 7:**
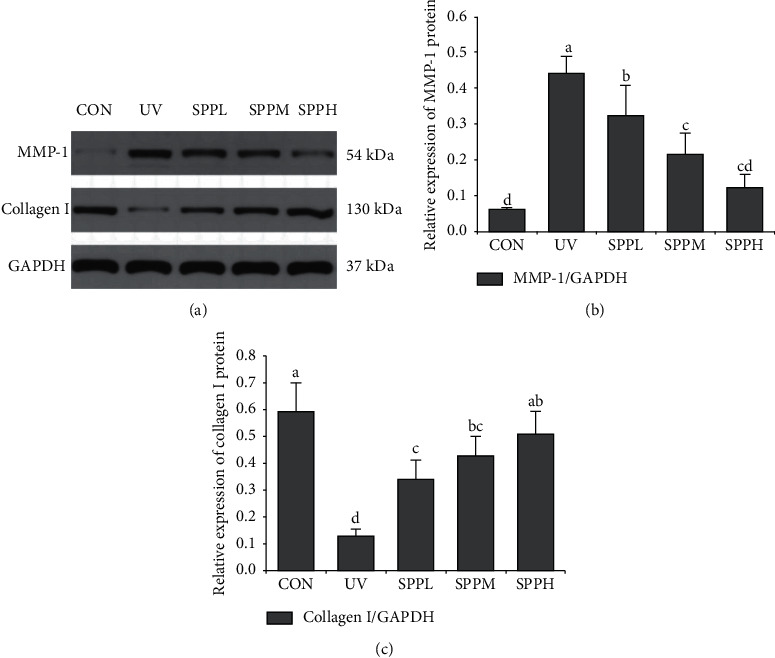
Effect of SPP on protein expression of MMP-1 (a, b) and collagen I (a, c) of photoaging skin in mice after the experiment; each bar represents the mean value from three repetitions with standard error (SE); different letters represent significant differences between the two groups.

Compared with the CON group, relative expression of MMP-1 protein in the skin of mice in the UV group increased significantly (*p* < 0.05). Compared with the UV group, relative expression of MMP-1 protein in the skin of mice in the SPPL, SPPM, and SPPH groups decreased significantly (*p* < 0.05). Compared with the SPPL group, relative expression of MMP-1 protein in the skin of mice in the SPPM and SPPH groups decreased significantly (*p* < 0.05) (Figures 7(a) and 7(b)).

Compared with the CON group, relative expression of collagen I protein in the skin of mice in the UV group decreased significantly (*p* < 0.05). Compared with the UV group, relative expression of collagen I protein in the skin of mice in the SPPL, SPPM, and SPPH groups increased significantly (*p* < 0.05). Compared with the SPPL group, relative expression of collagen I protein in the skin of mice in the SPPH group increased significantly (*p* < 0.05) (Figures 7(a) and 7(c)).

### 3.7. Effect of SPP on Phosphorylation Level of MAPK Signaling Pathway in Photoaged Skin of Mice

**Figure 8:**
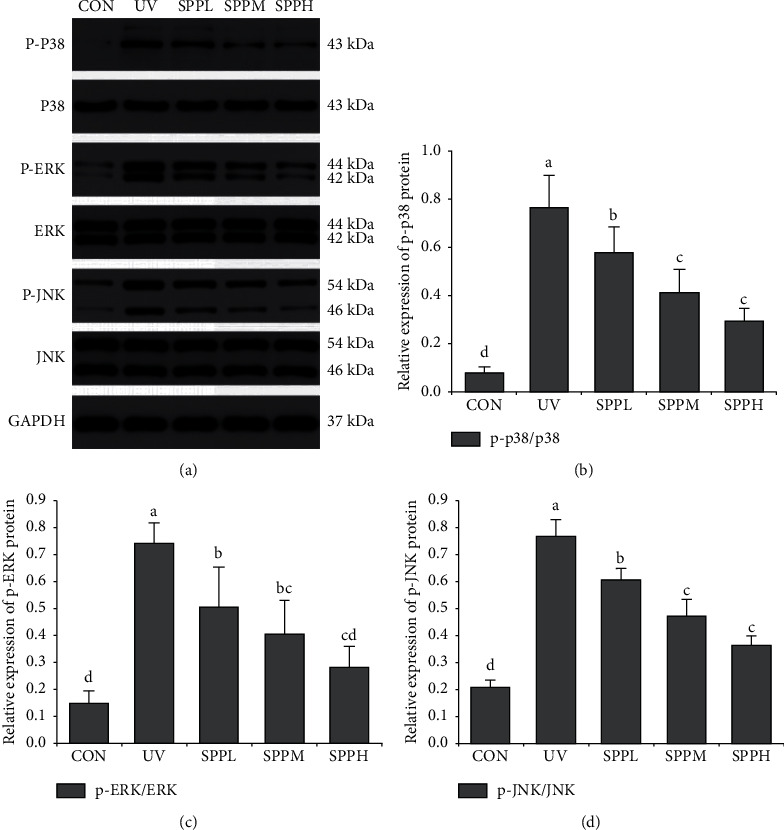
Effect of SPP on phosphorylation level of p38 (a, b), ERK (a, c), and JNK (a, d) of photoaging skin in mice after the experiment; each bar represents the mean value from three repetitions with standard error (SE); different letters represent significant differences between the two groups.

Compared with the CON group, relative expression of p-p38, p-ERK, and p-JNK proteins in the skin of mice in the UV group increased significantly (*p* < 0.05). Compared with the UV group, relative expression of p-p38, p-ERK, and p-JNK proteins in the skin of mice in the SPPL, SPPM, and SPPH groups decreased significantly (*p* < 0.05). Compared with the SPPL group, relative expression of p-p38 and p-JNK proteins in the skin of mice in the SPPM and SPPH groups decreased significantly (*p* < 0.05); relative expression of p-ERK protein in the skin of mice in the SPPH group decreased significantly (*p* < 0.05) (Figure 8).

## 4. Discussion

Increase in intracellular ROS levels is closely associated with cell senescence; thus, ROS scavenging ability is a potential target for prevention of aging [12]. Skin plays an important role in nonspecific immune mechanisms and serves as a major barrier against environmental insults [13]. UV-induced ROS overproduction is directly related to the occurrence of skin photoaging [14]. Skin photoaging results from disruption in the dynamic balance in oxidative stress through increased ROS production and decrease in the activity of antioxidant enzymes, such as SOD and CAT, which attack liposomes in skin cells and produce MDA, causing damage to the skin tissue structure [15, 16]. Thus, inhibition of UV-induced ROS production could be an effective strategy to prevent skin photoaging.

Medical texts affirm the medicinal value of pearl and state that its main functions are detoxification and muscle regeneration. In recent years, some scholars have shown that pearl extract has high antioxidant capacity *in vitro* and *in vivo*, which can prevent and treat diseases caused by ROS [17, 18]. Hence, we investigated the therapeutic effect of SPP on UV-induced photoaging in ICR mice. In this study, SPP downregulated ROS and MDA contents, upregulated SOD and CAT activities, and improved and restored skin appearance and tissue structure of UV-induced photoaging of skin in mice. The observed effects were dose-dependent in a certain concentration range, and the optimal concentration of seawater pearl powder was found to be 100 mg/g. Antioxidant enzymes like SOD and CAT cooperatively reduce ROS in the organisms [19]. Therefore, SPP has the ability to treat UV-induced skin photoaging in mice by scavenging excess ROS.

ROS overproduction activates the MAPK signaling pathway, which stimulates the transcription of MMP-1 genes in fibroblasts and negatively regulates the transcription of genes encoding type-I procollagen [20]. The results presented here show that UV irradiation could significantly upregulate MMP-1 expression and downregulate collagen I in the skin via activation of the MAPK signaling pathway, including ERK, JNK, and p38. Similar to our results, protein expression of MMP-1 increased and that of collagen I decreased by activation of signaling pathways in the human dermal fibroblasts and reconstructed skin model after UVB irradiation in a study [21]. Nevertheless, treating UV-induced skin photoaging with SPP significantly decreased the phosphorylation of ERK, JNK, and p38, which resulted in the attenuation of MMP-1 production and recovery of collagen I expression [22]. These results were dose-dependent in a certain concentration range, and the optimal concentration of seawater pearl powder was found to be 100 mg/g. Therefore, SPP has the ability to suppress MMP-1 expression and enhance collagen I production via inhibition of MAPK signaling pathway after scavenging excess ROS in UV-induced photoaged skin in mice.

Under normal physiological conditions, pigmentation protects the human skin from harmful damage caused by ultraviolet rays. However, excessive melanin can cause skin aging [23, 24]. Abundant production and excessive activation of TYR can cause excess formation of melanin [25]. The results presented here show that UV irradiation could significantly upregulate TYR activity and melanin content in the skin. Similar to these results, another study found that TYR activity and melanin content increased in human melanocytes and human skin after UV irradiation [26]. Nevertheless, treating UV-induced skin photoaging with SPP significantly decreased TYR activity and melanin content. These results were dose-dependent in a certain concentration range, and the optimal concentration of seawater pearl powder was 200 mg/g. Research indicates that ROS generated in the skin upon UV irradiation is responsible for melanin production, and effective antioxidants are recognized as useful tools for prevention of UV-induced melanin production [27]. Therefore, SPP has the ability to reduce melanin content via inhibition of TYR activity after scavenging excess ROS in UV-induced photoaged skin in mice.

## 5. Conclusions

SPP displayed significant therapeutic effect on photoaging skin in the form of improved and restored appearance and tissue structures in skin of mice. The major mechanism may be related to reduction of protein expression of MMP-1 and enhancing collagen I production via inhibition of signaling pathways (p38, ERK, and JNK) after scavenging excess ROS in UV-induced photoaged skin of mice. Meanwhile, it may also be involved in reducing melanin content via inhibition of TYR activity after scavenging excess ROS in UV-induced photoaged skin of mice. Therefore, SPP could be an effective substance to treat photoaging skin. Taking both cost-effectiveness and efficacy into consideration, the optimal concentration of SPP for treating skin photoaging could be 100 mg/g.

## Figures and Tables

**Table 1:** List of top 20 proteins in SPP detected by protein mass spectrometry.

No.	Majority protein IDs	Name	Mol. weight (kDa)	Sequence lengths	Intensity
1	A0A646RS43	Dmrt1	42.087	386	1676400000
2	A0A1S6R0G7	Sigma-class glutathione S-transferase	23.286	203	1367700000
3	A0A2P1H678	Alpha-1,4 glucan phosphorylase	100.67	874	986050000
4	J9PJ22	Arginine kinase	39.408	350	899540000
5	A0A2D1QTY0	Efetin	13.144	124	781770000
6	A0A6M8NXE9	Tyrosinase	81.7	730	653190000
7	A0A2P1H685	Elongation factor 1-alpha	50.66	462	589070000
8	A0EYM2	Glutathione peroxidase	26.379	232	446680000
9	A0A1Q1MMM3	Cytochrome c oxidase subunit 1	57.229	515	419940000
10	A0A1G5	Alpha-2-macroglobulin	180.28	1611	390670000
11	A0A6H1WVY6	Beta-actin	32.055	286	350140000
12	A0A1Q2HLX7	MORF	22.628	203	340990000
13	A0A2P1H679	60S ribosomal protein L8	29.586	272	339950000
14	A0A6M3W8I9	Complement component C3	188.4	1665	324990000
15	A0A5P1M9Z9	Beta-catenin	89.975	820	321470000
16	A0A2P1H677	Succinate dehydrogenase (ubiquinone)	32.042	288	300480000
17	A0A5J6SDR0	Transformaer-2 alpha	34.661	297	298430000
18	A0A2U8XEL1	Pif	112.08	1035	293310000
19	A0A2P1H676	Heat shock protein 70	74.999	686	276250000
20	A0A1P8AJ55	NADH-ubiquinone oxidoreductase chain 4	49.156	448	267720000

**Table 2:** Contents of trace elements in SPP (mg/kg).

S	P	Fe	Mn	Cu	Na	Mg	Zn	Sr	Se	As	Hg
746	74.3	26.3	9.66	2.03	5943	631	47.6	1003	0.046	3.50	0.0

## Data Availability

The data used to support the findings of this study are included within the article.

## Abbreviations

SPP: Seawater pearl powder
UV: Ultraviolet
ROS: Reactive oxygen species
MAPK: Mitogen-activated protein kinases
ERK: Extracellular signal-regulated kinases
JNK: c-Jun amino terminal kinase
AP-1: Activator protein-1
MMP-1: Matrix metalloproteinase-1
TYR: Tyrosinase
SOD: Superoxide dismutase
MDA: Malondialdehyde
CAT: Catalase.

## Supplementary Materials

Supplementary Materials See annexure 1 for list of 178 proteins in SPP detected by protein mass spectrometry.
