# Supplementary material for: Therapeutic Effect of Seawater Pearl Powder on UV-Induced Photoaging in Mouse Skin
Source: Evid Based Complement Alternat Med. 2021 Dec 9;2021:9516427. doi: 10.1155/2021/9516427 (PMC8677389; doi:10.1155/2021/9516427)
Supplement: Supplementary Materials — See annexure 1 for list of 178 proteins in SPP detected by protein mass spectrometry. [file 9516427.f1.doc]

**Annexure 1: List of 178 proteins in seawater pearl powder detected by protein mass spectrometry**

| NO. | Majority protein IDs | Name | Mol. weight [kDa] | Sequence lengths | Intensity |
| --- | --- | --- | --- | --- | --- |
| 1 | A0A646RS43 | Dmrt1 | 42.087 | 386 | 1676400000 |
| 2 | A0A1S6R0G7 | Sigma-class glutathione S-transferase | 23.286 | 203 | 1367700000 |
| 3 | A0A2P1H678 | Alpha-1,4 glucan phosphorylase | 100.67 | 874 | 986050000 |
| 4 | J9PJ22 | Arginine kinase | 39.408 | 350 | 899540000 |
| 5 | A0A2D1QTY0 | Efetin | 13.144 | 124 | 781770000 |
| 6 | A0A6M8NXE9 | Tyrosinase | 81.7 | 730 | 653190000 |
| 7 | A0A2P1H685 | Elongation factor 1-alpha | 50.66 | 462 | 589070000 |
| 8 | A0EYM2 | Glutathione peroxidase | 26.379 | 232 | 446680000 |
| 9 | A0A1Q1MMM3 | Cytochrome c oxidase  subunit 1 | 57.229 | 515 | 419940000 |
| 10 | A0A1G5 | Alpha-2-macroglobulin | 180.28 | 1611 | 390670000 |
| 11 | A0A6H1WVY6 | Beta-actin | 32.055 | 286 | 350140000 |
| 12 | A0A1Q2HLX7 | MORF | 22.628 | 203 | 340990000 |
| 13 | A0A2P1H679 | 60S ribosomal protein L8 | 29.586 | 272 | 339950000 |
| 14 | A0A6M3W8I9 | Complement component C3 | 188.4 | 1665 | 324990000 |
| 15 | A0A5P1M9Z9 | Beta-catenin | 89.975 | 820 | 321470000 |
| 16 | A0A2P1H677 | Succinate dehydrogenase [ubiquinone] | 32.042 | 288 | 300480000 |
| 17 | A0A5J6SDR0 | Transformaer-2 alpha | 34.661 | 297 | 298430000 |
| 18 | A0A2U8XEL1 | Pif | 112.08 | 1035 | 293310000 |
| 19 | A0A2P1H676 | Heat shock protein 70 | 74.999 | 686 | 276250000 |
| 20 | A0A1P8AJ55 | NADH-ubiquinone oxidoreductase chain 4 | 49.156 | 448 | 267720000 |
| 21 | A0A1S6R0H0 | Glutathione S-transferase A | 26.795 | 226 | 267130000 |
| 22 | A0A0F6WGC7 | Calcium-transporting ATPase | 131.64 | 1195 | 256680000 |
| 23 | A0A5B8XFI4 | Superoxide dismutase | 25.103 | 223 | 244050000 |
| 24 | A0A1P8AJ52 | NADH-ubiquinone oxidoreductase chain 2 | 35.567 | 321 | 223580000 |
| 25 | A0A5B7QC93 | Toll7 | 82.068 | 712 | 223040000 |
| 26 | A0A1Y0JYQ0 | TIMP-1 | 27.261 | 235 | 221240000 |
| 27 | A0A385H5C6 | Metal responsive transcription factor 2 | 53.793 | 466 | 209910000 |
| 28 | A0A4Y1KRB3 | ATP synthase subunit a | 25.254 | 230 | 194320000 |
| 29 | A0A1B2TRY4 | M-ORF | 10.851 | 96 | 193330000 |
| 30 | A0A5B7QCA4 | Toll6 | 86.118 | 740 | 189030000 |
| 31 | A0A346HGS3 | M-ORF1 | 22.225 | 200 | 188470000 |
| 32 | W8SQG8 | Heat shock protein 90 | 83.475 | 726 | 188030000 |
| 33 | A0A1Y0JYP0 | MMP-19 | 59.439 | 522 | 184340000 |
| 34 | A0A513X0E1 | Cytochrome b | 43.391 | 382 | 180440000 |
| 35 | V5QA07 | Cytochrome c oxidase subunit 2 | 45.435 | 407 | 162490000 |
| 36 | W8GTM5 | Heat shock protein 60 | 61.039 | 568 | 161970000 |
| 37 | A0A346HGW1 | NADH-ubiquinone oxidoreductase chain 5 | 64.932 | 585 | 160060000 |
| 38 | F6MIV7 | Cathepsin B | 38.552 | 347 | 147530000 |
| 39 | A0A2P1H674 | Glutathione synthase | 17.725 | 152 | 146400000 |
| 40 | R4VDM5 | Apolipophorin | 128.16 | 1155 | 142670000 |
| 41 | A0A2H4C3X6 | Y-box protein | 26.458 | 229 | 142560000 |
| 42 | A0A097A0B5 | Cyclophilin D | 41.094 | 367 | 133580000 |
| 43 | A0A2Z4MTA0 | E3 ubiquitin-protein ligase TRAF6 | 75.044 | 654 | 106100000 |
| 44 | A0A291RBW5 | TRAF3 | 64.517 | 562 | 103990000 |
| 45 | A0A2R3ZQC1 | Toll5 | 107.62 | 941 | 102670000 |
| 46 | A0A2R3ZQC2 | Toll4 | 106.68 | 931 | 101990000 |
| 47 | A0A291S6Z4 | TNF receptor-associated factor 2 | 64.135 | 560 | 101250000 |
| 48 | A0A513X0G0 | Cytochrome c oxidase subunit 3 | 28.668 | 257 | 89306000 |
| 49 | A0A2D1QTZ0 | Krichin | 15.635 | 148 | 87587000 |
| 50 | A0A2H4UL83 | Inhibitor of apoptosis 2 | 46.224 | 413 | 87466000 |
| 51 | F6LQK2 | Interferon regulatory factor 2 | 37.576 | 329 | 86540000 |
| 52 | B5ACH4 | Alpha2-macroglobulin | 195.13 | 1741 | 86073000 |
| 53 | A0A7M3SZ02 | Dmrt A2-1 | 42.582 | 385 | 83598000 |
| 54 | A0A2H4UL82 | Signal transducer and activator of transcription | 90.023 | 788 | 81917000 |
| 55 | A0A1Y0JZ53 | Mothers against decapentaplegic homolog | 47.28 | 418 | 81714000 |
| 56 | A0A3Q8UGQ0 | NF-E2-related factor 2 | 99.938 | 887 | 81509000 |
| 57 | A0A2H4UL81 | Inhibitor of apoptosis 1 | 64.299 | 573 | 78615000 |
| 58 | A0A1B2TRZ5 | HORF | 21.687 | 202 | 72658000 |
| 59 | A0A3G1GHW4 | NADH dehydrogenase subunit 6 | 27.08 | 245 | 71679000 |
| 60 | A0A343BS07 | Ankyrin repeat protein | 66.119 | 603 | 66199000 |
| 61 | A0A1J0KGN7 | Tyr | 75.814 | 662 | 65651000 |
| 62 | A0A650AWB1 | Matrix protein X | 15.063 | 133 | 65366000 |
| 63 | A0A140H909 | Catalase | 58.063 | 512 | 63982000 |
| 64 | Q5XQF8 | NaK-ATPase alpha subunit | 22.289 | 197 | 63254000 |
| 65 | A0A097A0B4 | Peptidyl-prolyl cis-trans isomerase | 22.986 | 210 | 62259000 |
| 66 | J9U877 | Calreticulin | 48.978 | 417 | 59274000 |
| 67 | H2DH25 | Cathepsin L | 37.74 | 333 | 58131000 |
| 68 | A0A1W5X9T4 | Nacreous layer matrix protein Hic52 | 54.291 | 542 | 54676000 |
| 69 | A0A1U8VAJ7 | NADH-ubiquinone oxidoreductase chain 1 | 67.521 | 596 | 52588000 |
| 70 | A0A1N7TAU5 | Phage lysozyme 1 | 21.282 | 190 | 50012000 |
| 71 | A0ASU4 | Cytochrome oxidase subunit II | 29.811 | 269 | 49705000 |
| 72 | Q5BTY5 | GST class-pi | 23.408 | 205 | 49453000 |
| 73 | A0A1U9WZ04 | Galectin | 17.156 | 149 | 48368000 |
| 74 | G0YWE5 | SRY-box containing protein 2 | 28.307 | 251 | 46932000 |
| 75 | A0A221SSF6 | GST2 | 24.939 | 213 | 46377000 |
| 76 | A0A6G6C403 | Interleukin-1 receptor-associated kinase 4 | 62.348 | 557 | 45906000 |
| 77 | K0I9M2 | 40S ribosomal protein SA | 33.134 | 300 | 44879000 |
| 78 | J7FHI0 | Chitin deacetylase isoform B | 74.272 | 645 | 44590000 |
| 79 | A0A1Y0JYR2 | Serine/threonine-protein kinase receptor | 58.248 | 517 | 44224000 |
| 80 | A0A516RMH6 | Suppressor of cytokine signaling-6 | 52.22 | 458 | 43926000 |
| 81 | A0A2K8C5H5 | NADH-ubiquinone oxidoreductase chain 3 | 12.667 | 114 | 43374000 |
| 82 | A0A343BS06 | Fem-1 A-like protein | 69.812 | 622 | 38461000 |
| 83 | C4P9M5 | Cytochrome P450 | 58.015 | 510 | 36651000 |
| 84 | J9UEN7 | Myosin essential light chain | 17.49 | 155 | 36120000 |
| 85 | A0A2R4LWB1 | Calmodulin-like protein | 15.123 | 134 | 35802000 |
| 86 | A0A161AT82 | Bone morphogenic protein 7 | 49.14 | 428 | 35277000 |
| 87 | A0A2R4PCY1 | Ferritin | 20.166 | 174 | 33567000 |
| 88 | A0A6M9BMA4 | Transformer-2 beta | 20.697 | 176 | 33181000 |
| 89 | A0A1Z1G774 | Matrix metalloproteinase 1 | 58.286 | 510 | 31840000 |
| 90 | A0A7M1IJU8 | CUB domain-containing protein | 37.871 | 333 | 30911000 |
| 91 | A0A2H4K9C8 | GTRPB4 | 17.525 | 144 | 30813000 |
| 92 | A0A1Z1G770 | Bactericidal/permeability-increasing protein 1 | 56.421 | 501 | 30803000 |
| 93 | A0A7L7YYC0 | FOXL2 | 46.093 | 404 | 30711000 |
| 94 | A0A2H4K9E8 | GTRPE5 | 15.451 | 150 | 30376000 |
| 95 | G0YWE4 | Doublesex and mab-3 related transcription factor-like protein | 42.568 | 389 | 28513000 |
| 96 | A0A6M3YBZ5 | Cytochrome oxidase subunit 1 | 24.501 | 226 | 28193000 |
| 97 | F4ZG80 | M-specific morf protein | 24.004 | 218 | 28156000 |
| 98 | A0A7G3FWG6 | Hic19 | 21.26 | 192 | 28075000 |
| 99 | L7PD08 | Metal binding protein | 12.762 | 117 | 27993000 |
| 100 | A0A2D1QUA7 | Tesein-1 | 21.409 | 194 | 27425000 |
| 101 | A0A7D7QIP9 | LIM/homeobox protein Lhx9 | 21.665 | 189 | 26685000 |
| 102 | A0A060ANB4 | Toll2 | 134.62 | 1158 | 24035000 |
| 103 | A0A2H4PM72 | MafK | 21.92 | 189 | 23598000 |
| 104 | D2DVZ8 | dehydrogenase subunit 6 | 19.607 | 173 | 22886000 |
| 105 | A0A0B5G2C0 | CUB domain-containing protein | 72.678 | 639 | 22612000 |
| 106 | A0A1L2FYM6 | Complement C1Q-like protein | 34.273 | 304 | 22231000 |
| 107 | A0A0K2BNB9 | Hic31 | 30.751 | 318 | 22150000 |
| 108 | A8UMW5 | Putative tumor suppressor QM protein | 14.428 | 128 | 21682000 |
| 109 | A0A6H0F2G7 | Hydroxymethylbilane hydrolyase [cyclizing] | 31.688 | 285 | 20824000 |
| 110 | A0A1J0KEL5 | Tyrp-1 | 87.582 | 778 | 20596000 |
| 111 | A0A346HGW7 | M-ORF2 | 22.418 | 200 | 19817000 |
| 112 | A0A161VF18 | Perlucin2 | 21.711 | 182 | 19573000 |
| 113 | A0A0P0M6J7 | Carbonic anhydrase | 38.271 | 332 | 19274000 |
| 114 | A0A2P1H671 | Citrate synthase | 55.056 | 496 | 19228000 |
| 115 | A0A0S1YBQ4 | Receptor for activated C kinase 1 | 35.364 | 318 | 18911000 |
| 116 | A0A1N7TAW0 | Phage lysozyme 2 | 17.787 | 155 | 17233000 |
| 117 | R4UY41 | Scavenger receptor cysteine-rich protein 2 | 31.957 | 278 | 17189000 |
| 118 | A0A2H4UL76 | Apoptosis inducing factor | 73.832 | 682 | 16585000 |
| 119 | A0A481S0K9 | Microphthalmia-associated transcription factor | 49.823 | 443 | 16184000 |
| 120 | A0A2P1H686 | Uracil-DNA glycosylase | 31.37 | 281 | 15918000 |
| 121 | A0A2S1ZUZ8 | F-specific ORF protein | 11.142 | 102 | 15835000 |
| 122 | A0A291NZU8 | Metal response element-binding transcription factor-1 | 57.4 | 516 | 15340000 |
| 123 | A0A2Z0PN28 | Phosphoglycerate mutase | 28.661 | 250 | 14864000 |
| 124 | A0A7U0FMC4 | NADH dehydrogenase subunit 5 | 62.294 | 578 | 14604000 |
| 125 | A0A1W5VMF2 | Hic74 | 73.702 | 850 | 14365000 |
| 126 | J7FIC1 | Chitinase-3 | 72.523 | 653 | 12946000 |
| 127 | A4UUE2 | Calmodulin | 15.131 | 135 | 12860000 |
| 128 | E1B4V3 | Lea/Rab family small GTPase | 24.919 | 221 | 12547000 |
| 129 | B4YE15 | Glutamate--cysteine ligase | 13.836 | 121 | 11743000 |
| 130 | A0A1I9RHH4 | Allograft inflammatory factor-1 | 16.713 | 147 | 11543000 |
| 131 | A0A1X9JUU4 | NADH-ubiquinone oxidoreductase chain 6 | 18.958 | 167 | 10541000 |
| 132 | M1S071 | Thioredoxin | 11.789 | 105 | 10507000 |
| 133 | A0A7G3FWH8 | Hic14 | 16.814 | 151 | 10150000 |
| 134 | A0A2H4K9G0 | Hicsilin 3 | 26.806 | 300 | 9658100 |
| 135 | F4ZG24 | H-orf protein | 27.38 | 248 | 9637800 |
| 136 | F4ZFI1 | Female-specific orf protein | 10.6 | 94 | 8883600 |
| 137 | A0A5J6KNP5 | ADP/ATP translocase | 17.14 | 155 | 8597300 |
| 138 | A0A1Y0JZ25 | Beta-arrestin 2 | 40.034 | 355 | 8354300 |
| 139 | A0A1Y0JYQ7 | Metalloproteinase inhibitor 4 | 16.589 | 150 | 8106800 |
| 140 | Q0G8K0 | High-affinity serotonin transporter protein | 21.696 | 189 | 7848400 |
| 141 | A0A3S8V9A9 | Kelch-like ECH-associated protein 1 | 68.332 | 612 | 7791400 |
| 142 | E2JEI4 | Peroxiredoxin 6 | 24.248 | 218 | 7746500 |
| 143 | A0A221SSE1 | GST1 | 28.242 | 248 | 7577900 |
| 144 | A0A1P8AFZ7 | M-specific ORF protein | 22.614 | 203 | 6984000 |
| 145 | A0A7G3FWI8 | Hic24 | 23.546 | 217 | 6874900 |
| 146 | A0A193DTC0 | SCD1 | 37.809 | 329 | 6667000 |
| 147 | A0A023I760 | Sigma class glutathione S-transferase | 23.336 | 203 | 6295300 |
| 148 | A8UMW1 | Glutathione transferase | 14.675 | 127 | 6162100 |
| 149 | A0A2U7PQK3 | F-ORF protein | 11.938 | 102 | 5637700 |
| 150 | A0A6M3YC52 | Histone H3 | 12.616 | 113 | 5443800 |
| 151 | A0A2H4K9D3 | GTRPB2 | 13.856 | 148 | 5357900 |
| 152 | B2MWP4 | Shaker cognate b isoform a | 19.982 | 177 | 5225900 |
| 153 | A0A023I1E6 | NADH dehydrogenase subunit 4L | 21 | 188 | 5097400 |
| 154 | D2KN89 | Arginase | 36.81 | 335 | 5052900 |
| 155 | A0A2L1K026 | Hichin | 20.367 | 180 | 5010300 |
| 156 | F4ZFF9 | F-ORF | 10.033 | 92 | 4890500 |
| 157 | F4ZFW7 | H-orf protein | 12.211 | 113 | 4718200 |
| 158 | J7FHX7 | Chitin deacetylase isoform A | 70.101 | 606 | 4106000 |
| 159 | A0A0N7J853 | Gastric intrinsic factor-like protein 2 | 16.976 | 148 | 4061800 |
| 160 | A0A2Z5ZC60 | Retinoid X receptor | 48.84 | 444 | 3963500 |
| 161 | A0A1L1ZYQ6 | Phage lysozyme 2 | 17.73 | 155 | 3593300 |
| 162 | A0A2H4K9E2 | GTRPC6 | 16.46 | 173 | 2938500 |
| 163 | A0A2R4LWB3 | Chitin synthase | 27.536 | 237 | 2918800 |
| 164 | A0A2R4LWB0 | Glyceraldehyde 3-phosphate dehydrogenase | 17.497 | 162 | 2787000 |
| 165 | K0P7H2 | Upsalin | 14.007 | 125 | 2756700 |
| 166 | A0A1Z1G775 | Bactericidal/permeability-increasing protein 2 | 56.742 | 518 | 2400300 |
| 167 | B8QE55 | Cytochrome c oxidase subunit 1 | 19.777 | 186 | 2388400 |
| 168 | A0A7U0IUS0 | NADH dehydrogenase subunit 2 | 35.409 | 321 | 2366000 |
| 169 | R4VGA5 | Scavenger receptor cysteine-rich protein 1 | 30.524 | 272 | 1660900 |
| 170 | A0A3G2KWM0 | Carbonate dehydratase IV | 34.086 | 294 | 1645600 |
| 171 | A0A0P0M6C9 | Gastric intrinsic factor-like protein 1 | 16.201 | 140 | 1486600 |
| 172 | A0A7L8G423 | ADP/ATP translocase (Fragment) | 17.197 | 156 | 1359500 |
| 173 | E1B300 | Thioredoxin peroxidase | 21.872 | 196 | 1019400 |
| 174 | Q6QWQ4 | Cytochrome P450 CYP4 | 11.856 | 104 | 851270 |
| 175 | B6E105 | Superoxide dismutase [Cu-Zn] | 15.791 | 155 | 637880 |
| 176 | A0A1S6R0G4 | Thioredoxin-like protein | 16.88 | 142 | 474420 |
| 177 | A0A1N7TAU8 | Phage lysozyme 3 | 17.802 | 155 | 448090 |
| 178 | R4H2U7 | L-type voltage-dependent calcium channel protein beta subunit | 15.732 | 144 | 385650 |
